# Rural Household Food Insecurity among Latino Immigrants during the COVID-19 Pandemic

**DOI:** 10.3390/nu14132772

**Published:** 2022-07-05

**Authors:** Denise Diaz Payán, Fabiola Perez-Lua, Sidra Goldman-Mellor, Maria-Elena De Trinidad Young

**Affiliations:** 1 Department of Health, Society and Behavior, Program in Public Health, University of California, Irvine, CA 92697, USA; 2 Department of Public Health, School of Social Sciences, Humanities and Arts, University of California, Merced, CA 95343, USA; fperez-lua@ucmerced.edu (F.P.-L.); sgoldman-mellor@ucmerced.edu (S.G.-M.); mariaelena@ucmerced.edu (M.-E.D.T.Y.)

**Keywords:** food security, Latino immigrants, rural health, nutrition, COVID-19

## Abstract

U.S. food insecurity rates rapidly increased during the COVID-19 pandemic, with disproportionate impacts on Latino immigrant households. We conducted a qualitative study to investigate how household food environments of rural Latino immigrants were affected during the COVID-19 pandemic. Thirty-one respondents (42% from low food security households) completed interviews (July 2020–April 2021) across four rural counties in California. A rural household food security conceptual framework was used to analyze the data. Early in the pandemic, food availability was impacted by school closures and the increased consumption of meals/snacks at home; food access was impacted by reduced incomes. Barriers to access included limited transportation, excess distance, and lack of convenience. Key resources for mitigating food insecurity were the Supplemental Nutrition Assistance Program (SNAP), the Pandemic Electronic Benefits Transfer (P-EBT), school meals, charitable food programs, and social capital, although the adequacy and acceptability of charitable food distributions were noted issues. Respondents expressed concern about legal status, stigma, and the public charge rule when discussing barriers to government nutrition assistance programs. They reported that food pantries and P-EBT had fewer access barriers. Positive coping strategies included health-promoting food substitutions and the reduced consumption of meals outside the home. Results can inform the development of policy and systems interventions to decrease food insecurity and nutrition-related health disparities among rural Latino immigrants.

## 1. Introduction

Food insecurity rates in the United States (U.S.) rapidly increased during the COVID-19 pandemic [1,2], with elevated rates in Black, Latino, and low-income households [3,4,5]. Food insecurity is associated with greater health care expenditures [6] and worse health behaviors and outcomes [7], including lower-quality diet [8,9,10], diet-related chronic illness (e.g., diabetes, cardiovascular disease) [7,11,12,13,14,15], and premature death [16,17].

The COVID-19 pandemic’s employment crisis exacerbated economic instability and food insecurity among marginalized populations. Economic impacts disproportionately affected U.S. Latino households [1,4], who faced a multitude of structural barriers that influenced their food security status and diet [18]. Latinos struggled with increased economic and health vulnerabilities during the pandemic since they were concentrated in lower-wage occupations [19], more likely to work outside the home, and less likely to obtain economic aid through stimulus checks or unemployment benefits [20,21], thereby increasing their risk of food insecurity.

Latino immigrants in rural communities may have uniquely experienced food shortages during the pandemic due to existing economic, social, and health vulnerabilities. Rural America has increasingly become an immigrant destination with a growing concentration of Latino immigrants [22]. However, inequalities like lack of employment, housing instability, limited access to health care services, and language access barriers can result in lower social status in rural areas [23] and increase Latino households’ food insecurity risk [24]. Limited public transportation and availability of other social/health care services [25] can further curtail food access [2].

Several government nutrition assistance programs, including the Supplemental Nutrition Assistance Program (SNAP, formerly food stamps) and free or reduced-price school meals, exist to alleviate food insecurity among low-income households in the U.S. During the COVID-19 pandemic, access to and utilization of these programs may have been impeded by shelter-in-place orders and school closures. Nutrition assistance programs (e.g., USDA) approved waivers and adopted flexibilities designed to facilitate access during the pandemic, such as allowing schools to be flexible with meal distribution while promoting social distancing (i.e., parents/guardians could pick up children’s meals without a child present) [26]. For Latino immigrant households, confusion about program eligibility or new distribution modalities, fear of discrimination, the chilling effect from public charge, and language access barriers [18,27,28,29,30] may have reduced access to and use of these services [26,31].

Research to document and understand the impacts of the COVID-19 pandemic on food security elements and coping mechanisms is limited [32], particularly in rural communities [1]. Despite the profound effects of the pandemic on the health and economic well-being of Latino immigrants, little is known about their experiences with household food insecurity or how they coped during this period [18]. Qualitative research can reveal the lived experiences and coping strategies undertaken by food-insecure households and help to elucidate the mechanisms underlying the relationships between structural conditions, food insecurity, and health [33].

This article investigates how the household food environments of Latino immigrants in rural communities were impacted during the COVID-19 pandemic. We were particularly focused on understanding how nutrition assistance resources (e.g., SNAP, school meals, charitable food assistance) may have helped to alleviate food insecurity during the pandemic. These findings can inform the development of policy, systems, and environmental interventions to decrease food insecurity and nutrition-related health disparities for Latinos in rural regions and beyond.

## 2. Materials and Methods

This qualitative study used in-depth, semi-structured interviews to collect data and a conceptual framework of rural household food insecurity to guide analysis. The parent study [21] used a community-engaged research approach, which seeks to integrate minoritized voices and communities of color as study collaborators [34], by partnering with federal, state, and local immigrant serving organizations who participated on a community advisory board.

### 2.1. Data Collection

Recruitment relied on a convenience sample of Latino immigrants. The eligibility criteria consisted of: being (1) a minimum of 18 years old and (2) born in Mexico or a country in Central America and (3) living in a rural or agricultural community with a population <50,000 in one of four counties (Imperial, Merced, Tulare, or Fresno) in California. These four counties were selected because they were identified by advisory board members as having rural-identifying communities with large Latino populations, confirmed by our review of county demographic data that verified that each county contained numerous non-metropolitan areas with towns and small cities (≤50,000 population). Three counties were located in the San Joaquin Valley with the fourth in the Imperial Valley (Imperial County).

The advisory board referred interested individuals to the study. Individuals not referred by the board were recruited through social media posts or the research team’s personal networks. Interested individuals were assessed for eligibility. Eligible individuals provided verbal consent and received a $25 e-gift card. Study procedures and protocols were approved by UC Merced’s Institutional Review Board.

Data collection materials included an interview script with open-ended questions to obtain information about: household-level economic impacts and concerns during the pandemic, COVID-19-related stressors, food insecurity experiences, household food access and acquisition, use of nutrition assistance and social welfare programs, physical and mental health needs, social support, and health care access. Participants were specifically asked to describe any changes to their diet/household meals and food acquisition habits, identify challenges to purchasing or obtaining food, and detail their experiences with school meals (if they had children) during the pandemic. They also answered a brief survey with questions on sociodemographic and household characteristics (gender, age, nativity, length of residence in the U.S., employment status, education, marital status, household size, income), health issues, and government assistance program participation. The survey included the USDA’s six-item short food security module to minimize respondent burden [35].

Recruitment and data collection instruments were available in English and Spanish. A graduate student researcher and the principal investigator, both fluent in Spanish, translated the interview instrument and survey questions that were not already available in Spanish. Bilingual researchers and assistants fluent in Spanish reviewed and finalized the tools with minor modifications after the first set of interviews.

A trained, bilingual research staff member conducted all the interviews between July 2020 and April 2021. For confidentiality purposes, no actual names were used, and pseudonyms were assigned to each participant.

### 2.2. Data Analysis

The interviewer took detailed notes during each interview and completed a detailed memo with key themes afterward. Interviews were audio recorded with permission (duration range: 30–127 min) and transcribed verbatim in their original language. Transcripts were uploaded to Dedoose, a data management software program [36].

During data collection, the research team regularly met and discussed whether theoretical saturation had been achieved for the principal economic and health themes and to document emergent findings. Staff used a combination of inductive and deductive methods to identify principal themes and codes [37]. First, we used a grounded theory approach, which involved line-by-line coding to develop an initial list of codes. Three staff members piloted the draft codebook with six transcripts to test the initial coding structure and for comparison before using the final version to complete all coding. Two staff members met with the principal investigator on a weekly basis to discuss and resolve any discrepancies. Additional details about this analytical phase are available elsewhere [21].

The lead author used an existing conceptual framework to complete data analyses. Piaskoski, Reilly, and Gilliland developed a conceptual model of rural household food insecurity based on a systematic review of related qualitative research in the rural areas of developed countries. Key themes included five core food security elements (availability, accessibility, acceptability, adequacy, agency) in addition to four interacting themes for food-insecure households in rural areas: human capital, social capital, coping with compounding stressors, and navigating complex systems [38]. The lead author used these concepts to further analyze already coded output and other transcript data. After coding eight transcripts, data saturation was confirmed for these codes.

Preliminary findings were discussed and interpreted with the entire team. Illustrative quotations were selected for inclusion in this article, and Spanish quotations were translated to English by bilingual staff. Descriptive statistics were computed using Stata 16.1 [39].

## 3. Results

### 3.1. Participant Characteristics

A total of 31 respondents completed an interview (average age = 45 years; 65% female). A majority (77%) were married/lived with a partner, with an average household size of five. Most were born in Mexico (87%) and had lived in the U.S. for an average of 24 years. A majority (71%) had obtained a high school education or less, and nearly a quarter reported a chronic illness diagnosis.

A total of 42% were from households with low or very low food security (i.e., food-insecure households), while 26% were marginally food secure. While 71% (*n* = 22) reported participating in the state’s Medicaid program, less than half (48%) reported participating in SNAP. Only 16% had ever participated in the Special Supplemental Nutrition Program for Women, Infants, and Children (WIC) program. See Table 1.

### 3.2. Food Availability

Having more people physically present at home during weekdays was a key factor that reduced household food availability. Respondents said remote work and virtual schooling—in addition to fears of contracting COVID-19 outside the home—led to the rapid depletion of food due to increased snacking and consumption of meals at home.

Latino families with school-aged children described experiencing food shortages due to school closures and increased household food expenses. Several said that school meals had been critical pre-pandemic resources they relied on to offset food insecurity. Susana (very low food security, Tulare County) described this tension:

[Pre-pandemic] *they would receive free breakfast and lunch at school. When the children were home, they would eat more of everything. It’s more food, more milk, more everything. When they attended school, we would save* [money] *on food and now, well you can imagine, with two children at home all day. They are eating here and we are without work.*

Food availability was also impacted by supply chain interruptions and the reduced availability of specific items among retailers. Rosa (high food security, Merced County) described these shortages and the impact on her family’s diet:

*We tried to limit ourselves to what we could eat because the most important thing is to survive. Right? Even if it was only beans every day. That is a win for the stomach, but sometimes, there were no beans, no rice in the stores.*

### 3.3. Food Accessibility

Several respondents said reduced household income and lost wages influenced access. Pandemic food purchasing decisions were described as largely driven by household income reductions and the increased consumption of meals/snacks at home. Castro (marginally food secure, Merced County) said, “It was disappointing for me. Costs increased. Household costs increased because now there were five [people at home]”.

Most who obtained SNAP benefits said government assistance programs helped alleviate food costs during the pandemic (see 3.6. Food Agency). Some, however, thought the monthly allotment was insufficient to assist with increased costs. Victoria (low food security, Fresno County) remarked, “We have food stamps as well. But they do not give you very much and sometimes you do not have enough for food”. Mimi (low food security, Fresno County) similarly commented, “It was difficult to pay the bills and buy food. We had help from EBT, the food stamps, but it wasn’t enough for everyone who is home”.

Many respondents said they shopped at large discount stores, big box stores, and discount grocery stores to address affordability. Choco (high food security, Imperial County) spoke about the importance of frequenting less expensive stores:

*I go to the most inexpensive* [store] *now. I go to the 99-cent store, I go to Walmart and buy the cheapest avocados. I do what I can… with $10, I can buy food for the entire day… I buy many inexpensive items there at the 99-cent store. There are salads and a majority of items are 99 cents. I buy cheap bread and everything I need.*

A few said they exclusively shopped at discount retailers during the pandemic.

Latino immigrants in rural communities mentioned physical challenges to food access, including transportation and distance. Individuals in food deserts, where a grocery store was not available nearby, emphasized the importance of planning and budgeting gasoline, time, and other transportation costs beforehand. While small retailers were identified as important food sources, they had limited variety or were more expensive. Rogelio (low food security, Tulare County) described these challenges:

*If you do not plan ahead, you have to pay more. Salt, an egg, oil, all of that is expensive… and how much will you pay for gas? You have to plan ahead. Because you are far away from large stores and if you need to go to Walmart, you have to drive and it is far away. The liquor stores, the gasoline station stores, they are much more expensive.*

Parents also mentioned physical challenges to accessing school meals during closures. Transportation barriers and competing responsibilities were key issues. Lily (marginally food secure, Fresno County) said, “We never went to pick up school meals because it was too early, and I had to go work”.

Legal status was also mentioned as an impediment that limited economic opportunities and rendered many ineligible for government assistance programs. Some Latino immigrants spoke about the detrimental effects of being undocumented on their ability to obtain employment or income. Juan (marginally food secure, Fresno County) described ongoing worries experienced during the pandemic that were coupled with fear of contagion: “Like those without papers. They worry, ‘How will I work if I get sick? How will I pay my bills? How will I purchase food for my children?’ These are huge concerns”.

### 3.4. Food Adequacy

Concerns about the adequacy of food distributed by charitable assistance programs were raised. Food pantries were described as having limited availability of more nutritious, fresh produce items, and, instead, had higher quantities of canned food items. Expired food was also an issue. Mimi (low food security, Fresno County) explained that some items were expired, which could lead to waste, “In reality, it helped us a great deal but the powdered milk, the canned food, some of it was expired and well, we couldn’t use them, so we threw them away”.

### 3.5. Food Acceptability

Children’s food preferences emerged as an important food acceptability theme. Latino parents, especially mothers, described prioritizing the purchase of food that was more likely to be consumed by their children. Eli (high food security, Tulare County) shared:

*I began to pay more attention to what the children ate. I asked them, “what do you like?” “Not this, mom.” I tried to have sufficient food that they would eat, that they would not waste… I bought the girls things like Cheetos, juice, snacks, that they would eat.*

Juana (very low food security, Fresno County) recounted experiences where her children’s preferences were the basis for decisions about procurement and preparation, “That’s what they like the most—the tamales, the pizza, the hamburgers. If I didn’t get those things, if I said no, then they would get mad”.

Children’s food preferences also influenced school meal access early in the pandemic. Several parents said they did not prioritize picking up meals or ceased doing so due to their children’s dislike of school meals. Gladis (high food security, Fresno County) described the difficulty of balancing food availability with her children’s preferences:

*Every day, it has milk and fruit. In the morning, they give cereal and sometimes pancakes or waffles. For lunch, they have bagged lunches. Like burritos, orange chicken with rice… sometimes pizza. The reality is that sometimes children are very picky. I stopped going to pick up school lunches because it bothered me that the children would not eat them on a regular basis… I told my three girls I was no longer going to go because they would not eat it.*

Specific to charitable food assistance programs, some respondents spoke about the misalignment between food pantry items and cultural or household preferences. Some said there were items, like canned food, disliked by family members. Juana (very low food security, Fresno County) said, “I like [donations], especially what they give so you can prepare food. Like the rice, the beans… but not the canned food. Not really. We don’t really like it”. Cristina (very low food security, Merced County) commented that her household adapted to items that were less culturally acceptable in her household:

*We have adapted and eat what is given to us. It is very different… we have had to modify some things. Sometimes they give us bread but we are not used to the type of bread they give us. I don’t know, it is a different kind of bread given out by the churches. We have had to adjust some of our habits. Well, Latino food in particular, it is tortillas, salsas, sometimes red meat or chicken. We have had to change our food for canned food, for sandwich bread and well, a majority of the items are canned.*

### 3.6. Food Agency

Respondents primarily spoke of the importance of the following nutrition assistance programs during the COVID-19 pandemic: SNAP, Pandemic Electronic Benefits Transfer program (P-EBT), school meals, and charitable food assistance (e.g., food pantries).

#### 3.6.1. Supplemental Nutrition Assistance Program (SNAP)

SNAP, known in California as CalFresh, was often called “estampillas” (food stamps) by Latino immigrant respondents, who described it as an invaluable source of nutrition assistance during the pandemic. SNAP recipients said the program greatly helped to address household food insecurity and alleviate the strain of other costs. Karina (low food security, Fresno County) detailed the program’s importance and impact:

*We applied—well, my husband applied—to CalFresh. Thank God, it has been of great help. At least when it comes to food, the CalFresh we are receiving covers that… the other costs, like rent, we are saving little by little for that because we do not want to fall behind. My husband is unemployed right now and they haven’t sent him anything.*

Maria (marginally food secure, Merced County) described how her household benefited because her daughter and grandchildren were eligible, “My daughter and her girls, they get stamps. With that, we all eat. They give her $500, $530 in stamps. With that, we all eat. And then we can save our money”. A few mentioned the temporary pandemic-related SNAP benefit increase, saying it was very helpful.

#### 3.6.2. School Meals and Pandemic EBT (P-EBT)

Several parents, mainly mothers, described the value of school meal flexibilities and distribution during the COVID-19 pandemic. In terms of frequency, some said they went daily, whereas others would pick up batches of school meals on a weekly or biweekly basis. Many mentioned the importance of P-EBT, food program benefits offered during the pandemic to children in the free or reduced-price school meal program. Lily (marginally food secure, Fresno County) described the program: “My daughter, they gave me $200 and something, I think. She wasn’t going to school, so they sent her a card”. Respondents said P-EBT particularly helped to offset higher costs associated with having children at home. Several expressed gratitude for the provisional program, and some said it was the only form of government assistance they obtained during the pandemic. Rogelio (low food security, Tulare County) said the “card sent by the school for the children” was of considerable aid, particularly since his family was ineligible for stimulus funds and his wife ceased working. Daniel (very low food security, Tulare County) said his mixed-status family was ineligible for other forms of government assistance:

*Right now, the children eat more because they are at home. We are spending more money on everything. We did get help with these blank cards and it helped. It was like $360 for each child under the age of 18, and we did not qualify for the other help… since we do not have documents. The children were born here, but we did not qualify for the other $500.*

Claudia (low food security, Imperial County) spoke about P-EBT’s importance for her large household, particularly because they did not qualify for CalFresh:

*The money sent to us for food stamps, that was a big help because my kids can eat. I have three—four teenagers and my little one can eat too. They go through food faster than I can bring it home. So, it did help a lot… we’re in the middle of that threshold. We don’t qualify* [for CalFresh]*. We miss it by a couple of bucks.*

#### 3.6.3. Charitable Food Assistance

Charitable food assistance was frequently mentioned as a crucial resource during the pandemic to help meet basic needs. Karina (low food security, Fresno County) said donated food boxes were of great aid: “I get vegetables and do not have to buy them. It is a huge help for me. And milk, they sometimes distribute milk, yogurt, cream”. Cristina (very low food security, Merced County) shared the importance of aid during food shortages: “I have been without food because I do not get [SNAP]. Instead, I go the Salvation Army, Catholic Charities, and places that give out food”.

Others frequented multiple community sites to reduce food purchasing. Unlike housing or utility costs, food was described as a flexible expense because of the availability of charitable food aid. Juana (very low food security, Fresno County) described this impact:

*They give out food boxes and they give us milk. They also gave me mozzarella cheese, meat, potatoes, yogurt. It’s great. At the other place, they gave me a bottle of oil, flour, beans, and rice. Sometimes they give out turkey, ham, or chicken. Canned tomatoes… juice, apples, oranges. The truth is, it has been really helpful towards reducing our costs… then I only have to buy things like chili, pork, or red meat.*

Susana (very low food security, Tulare County) explained how these resources helped her household budget: “Right now, there are a lot of food donations available. We go frequently to see where they are giving out food to save at least a little bit of money on food costs. And it helps us to then pay a bill, like the electricity bill or gas”.

Respondents mostly mentioned church-based food pantries as primary food aid sources. Choco (high food security, Imperial County), who obtained staples from a church said,

*There is a church in Calexico who gives me many items, like a box with cheese, milk, a variety of items. It all helps… they also give out canned fruit, cheese and beans, other canned items. Many things, it’s like $20 or $30 in groceries and it all helps.*

A few mentioned specific organizations. Eli (high food security, Tulare County) described the content of boxes distributed by an organization that seemed to offer more fresh food compared with others, “Unión de Campesinos distributed food in boxes… even chicken came in a box… bags of apples, pears, and chopped pineapple… bags of rice, lentils, beans—the box was full”.

Some respondents went in person to pick up items while others mentioned drive-through distribution models that emerged during the pandemic where a box/items were placed in a car’s trunk. This process was said to be less stigmatizing and safer; however, it presented a barrier for those without access to a private vehicle.

Food pantries were seen as a useful source of aid with fewer access barriers compared with government assistance programs. Roberta (low food security, Tulare County) explained how community sites, like churches, were more accessible:

*Each Friday, they give out food. I think it is based on donations they receive because sometimes they give out meat and other times, fruits and vegetables. It is very good. We like to go there because they do not ask for anything in return—no identification or cards, nothing. You just go in your car, and they place the food in your car. You give your name, where you live. Also how many members are in your household. That’s it.*

### 3.7. Living with Household Food Insecurity in Rural Areas

Next, we provide results describing the lived experiences and coping strategies of Latino immigrants in rural regions when faced with food shortages and insecurity. We present findings focused on social capital, compounding stressors, and navigating complex systems.

#### 3.7.1. Social Capital

Several respondents spoke about the importance of social networks for material resources and social support to buffer food insufficiency during the pandemic. Obtaining assistance from family members, friends, and neighbors was described as pivotal. Luis (marginally food secure, Fresno County) mentioned the importance of sharing food: “The secret is to help one another and share with my brothers and all of that. That’s the secret—to help one another”. Some also described obtaining or redistributing food from charitable programs based on relative need. Rogelio (low food security, Tulare County) described how his cousin brought him donated food:

*My cousin goes to the church, her church, and she brings me a box of vegetables every so often. Not always, but maybe like once a month, she will bring be a box of fruits and vegetables… it’s of great help. The kids are here at home, and they spend the day wanting to snack on something, so they eat fruit. It has been such a help.*

In terms of redistribution, Rosa (high food security, Merced County) said:

*If they gave me a box of food and if I did not feel that I needed all of it, then I would give the box to another person who really needed it and give them that help… if you can help someone else, why not do it? Right? If they give me potatoes, then I am going to share.*

The availability of homegrown produce and agricultural products was also described as an important resource. Lily (marginally food secure, Fresno County) mentioned how items were exchanged:

*An advantage of living in the Central Valley is that we can obtain many things for free or from neighbors… we support each other. We speak and are friendly and if I have something to give them, I give it to them because it helps a lot during difficult times like these.*

Cristina (very low food security, Merced County) further shared how reciprocal exchanges could contribute to increased dietary diversity: “For example, we exchange food with neighbors. The other day, I took them a box of tomatoes and they gave me plums, a bag of plums”.

Social networks also served as an important source of information. Individuals spoke about the importance of distributing information about charitable food assistance programs (i.e., location, date). Jesus (marginally food secure, Tulare County) explained, “Sometimes it is friends who advise you where to go to obtain food. Then you go and it helps a great deal”.

#### 3.7.2. Coping with Compounding Stressors

Respondents mentioned a variety of compounding stressors and coping strategies employed when describing their experiences with economic insecurity and food shortages. Table 2 includes a list of key findings on compounding stressors and related coping strategies with illustrative quotations.

Fear of COVID-19 infection was a factor that influenced food acquisition behaviors early in the pandemic. Some respondents reduced the frequency of trips to food retailers and restaurants to reduce exposure. Others mentioned taking specific measures on trips, for example, by going early in the morning to avoid crowds or using protective gear to reduce infection risk.

Several said they modified procurement habits to address high costs and/or COVID-related economic insecurity. They substituted more costly foods with less expensive items, reduced portions (particularly meat consumption), increased meal preparation at home, and reduced out-of-home food consumption to cope with strained budgets. Respondents generally reported eating out less at restaurants and fast-food chains in the early months.

Specific to food substitutions, some of these changes led to the purchase of inexpensive, less nutritious items, while some respondents reported more nutritious household dietary habits. For the latter, examples include the substitution of sugar-sweetened beverages (e.g., soda, juice) with water or shifting from high-calorie, high-fat snacks to whole foods and grains that were said to be less expensive and more economical for households.

Some respondents who lived in food deserts said they purchased more in bulk and froze food to save money, reduce transportation costs, and reduce the number of trips to distant retailers.

#### 3.7.3. Navigating Complex Systems

Immigration-related policy exclusions and stigma emerged as key barriers to Latino immigrants’ access to government nutrition assistance resources. Respondents described fear, lack of knowledge, and uncertainty about the public charge rule as issues. Cristina (very low food security, Merced County) said:

*If we receive help from the government, they can classify us as a public charge due to our legal status. We’ve never obtained aid because we have that fear. No food stamps, none of it. We only go where documentation is not requested or where they ask for the most basic information… I know there is a way for my children to eat better, but I do not want to risk it.*

Juana (very low food security, Fresno County) similarly mentioned that her household did not receive nutrition assistance for this reason:

*I would like to get food stamps, but no. I say no because I would like my son to have the opportunity to submit an application* [for legal status]*. With Trump saying that everyone is a public charge, right? I have previously obtained assistance, the Medi-Cal program. Food stamps though, no. It has been many years since I have avoided it because I do want to see if my son can fix his* [legal] *documents because, well, we have no documents.*

## 4. Discussion

This article investigates how household food environments of Latino immigrants in rural communities in California were impacted during the COVID-19 pandemic. We found that reduced household incomes combined with having more family members at home strained already restricted budgets and impacted food availability. Those with school-aged children were especially vulnerable to food shortages due to reduced access to school meals. Mothers described the importance of balancing sufficient food availability with children’s food preferences, which leaned towards higher-calorie, less-nutritious foods. These findings are aligned with research conducted during the COVID-19 pandemic that found that families generally increased their reliance on high-calorie snack foods and desserts/sweets [40]: a troubling trend given rising childhood obesity trends [41]. Since household food insecurity can have detrimental dietary and health impacts on children [42,43,44], population studies are needed to examine the net effects of these patterns in at-risk groups during the pandemic.

Our findings indicate that key barriers to food access consisted of higher food costs in small retailers, lack of transportation, and distance to grocery stores, which have previously been identified as impediments to food security in rural areas [38,45]. We further identified that transportation was a barrier specifically for accessing school meals during the pandemic, which is also mentioned in a COVID-19 study conducted with San Joaquin Valley school district stakeholders [46]. Providing incentives for informal transportation networks [45], coordinating school meal deliveries [26], using community pick-up locations [46], and expanding public transit to nutritious retailers are strategies to mitigate transportation barriers in rural communities.

To extend food budgets during the pandemic, respondents shifted to low-priced discount stores like dollar stores, which are a category of discount chain stores in the U.S. that often sell staple foods (e.g., Dollar General, Family Dollar, Dollar Tree stores). Since 2000, dollar stores have considerably expanded throughout the U.S.; empirical evidence suggests that once they enter a low-income neighborhood, they may not improve food access [47]. Rural customers rely more on mass merchandisers (e.g., Walmart) and dollar stores for packaged food purchases than other retailers and purchase more calories per person per day at these sites compared with urban counterparts [48]. Data from Ohio reveal the potential resilience of dollar stores during an economic downtown since visits to dollar stores declined the least compared with other retailer types during the pandemic [49]. Future work should investigate the impact of dollar stores on food access and diet in low-income and rural communities.

We found that food pantries, particularly church-based sites, were key external agencies used by rural Latino immigrants to mitigate food shortages during the pandemic and had fewer access barriers compared with government programs. Respondents mostly spoke in satisfied terms about donated items like fresh meat, fruits, and vegetables as well as culturally relevant staples. Our data, however, suggest that food adequacy and acceptability were issues in food pantries, shedding light on important yet understudied dimensions of food security [38]. Items distributed by food pantries may be of poor nutritional quality [50,51,52,53] and not adhere to clients’ cultural and dietary preferences [54,55], including in rural communities [56]. Studies are needed examining the nutrition environments of emergency food aid organizations during the COVID-19 pandemic.

For Latino households, immigration concerns and legal status can make access to nutritious meals even more difficult [24,57] and discourage individuals from participating in safety net programs. Respondents in our study spoke about the detrimental effects of being undocumented on their ability to obtain gainful employment and income or access government nutrition assistance. Some mentioned the public charge rule as an impediment to SNAP enrollment, adding empirical evidence to support concerns about the rule’s chilling effect on public assistance during the COVID-19 pandemic [26]. During the pandemic, there is evidence of inequitable SNAP utilization and expansion, namely reduced participation among low-income Latino households and a limited impact of SNAP expansion on minority households and those experiencing job loss [5]. While our study did not collect data on experiences with discrimination as a potential food access barrier, this is an important construct to assess in future work since racialized minorities can experience discrimination in a variety of settings, including food retail stores [51].

Children’s food preferences were an important food acceptability theme for school meals. Pre-pandemic, lack of variety or limited quantities of preferred food items were identified as barriers to school meal consumption among Black and Latino urban high school adolescents [58]. Choice and variety were likely more limited during school closures. Expanding fresh food options [46,58], increasing meal reimbursement rates, updating nutrition standards, and promoting farm-to-school programs in rural areas can help to increase participation and bolster nutritional quality.

Results also highlight the vital importance of school meals for improving food availability among Latino immigrants in rural communities during economic crises. In the U.S., the National School Lunch and School Breakfast Programs are federally assisted meal programs whereby participating schools receive reimbursement for meals served to students who qualify. Adopting a permanent nationwide universal free school meal program is a policy strategy to counter rural food insecurity in the U.S. and may also reduce administrative and eligibility barriers for immigrant and low-income families. P-EBT was especially praised by Latino parents in our study since it offered multiple benefits like offsetting increased food costs, allowing the purchase of preferred items, and expanding access to children whose families who might not otherwise qualify for government assistance. Implementation research examining the reach and utilization of P-EBT and school meal modifications across racial/ethnic groups, socioeconomic classes, and urban–rural settings is needed to identify areas for improvement.

Similar to comparable work elsewhere [2,33] including with Latino households [55], our sample of rural respondents spoke about the importance of social networks for material and social support to mitigate experiences of food insecurity. Reciprocity, trust, and access to homegrown agricultural food products were mentioned as factors underlying individual-level receipt or redistribution of food aid. Social capital has been shown to be positively associated with household food security in low-income households, including rural communities [38,59], and can reduce the risk of hunger [60]. Micro-pantries that promote the within-neighborhood reciprocity of food resources are an informal strategy that does not rely on existing social relationships [61]. Mutual aid organizations may also be a promising vehicle to help address food insecurity since they: promote a mutually beneficial reciprocal exchange of services and resources, rely on a shared power distribution model, and advance food sovereignty in local communities [62].

We identified health-promoting coping behaviors that may have improved the diet quality of Latino immigrants during the COVID-19 pandemic. Positive coping behaviors included the reduced frequency and consumption of meals outside the home and an increased preparation of meals at home. Survey research with U.S. households found that a majority of parents reported decreasing consumption of takeout/fast-food/already prepared meals and increasing home-cooked meals [40]. Our study further reveals that limited household budgets and concerns about SARS-CoV-2 infection may have contributed to these changes. Increased cooking at home may have been associated with improved diet quality early in the pandemic [63] since cooking and sharing meals at home has many benefits, including a more nutritious diet and healthier outcomes [64,65,66], while eating outside the home—particularly at fast-food outlets—is associated with lower-quality diets and obesity [67]. It is unclear whether these modifications were sustained or if positive effects were diluted with the increased consumption of high-calorie snack foods and desserts/sweets [40]. Further, it is important to note that gendered expectations about family roles (particularly around childcare responsibilities and food preparation) may have disproportionately impacted Latina mothers during the pandemic [18].

Some respondents said food substitutions could lead to healthier eating, including substituting sugar-sweetened beverages with water. Increased water intake and reduced sugary drink consumption was mentioned in a Minnesota study that surveyed emerging adults during the pandemic [51]. Health-promoting substitutions and shifts in food procurement and preparation are possible mechanisms to help explain puzzling results from a national-level study that found diet quality significantly improved during the Great Recession when unemployment levels were highest; however, these improvements were not maintained and had reversed by 2017–2018 [68], which raises important questions about the sustainability of any health-promoting coping strategies we identified. A replication study on the impacts of the economic recession on diet quality during the COVID-19 pandemic would help to elucidate trends.

Study strengths include our use of a community-engaged research approach and recruitment of highly marginalized individuals who are immigrants, speak Spanish, and are from food-insecure households during the pandemic. Participants in rural communities and from marginalized groups may be more reluctant to participate and are highly underrepresented in studies. It is possible some respondents were more comfortable with participating due to the convenience and confidentiality afforded by remote data collection [69]. Methodological strengths include the use of a rural household food security conceptual framework to guide data analysis, the use of two types of saturation to inform analytical decisions, and a sample size above the range of 9–17 interviews that has been identified as adequate for obtaining saturation based on a systematic review [70]. The study period also allowed for a sufficient lag time for the implementation of COVID-19-related policies and program modifications.

Limitations include limited external validity due to sampling methods used, the lack of items specifically focused on rural household food security elements in the data collection materials, and the lack of follow-up with participants to understand their longer-term experiences during the pandemic.

## 5. Conclusions

Household food environments in rural communities were impacted during the COVID-19 pandemic. This study details how Latino immigrants in rural California experienced and contended with food insecurity during this precarious period, noting key barriers to government nutrition assistance programs that may be unique to some immigrant groups. The results provide timely and policy-relevant evidence to improve safety net program implementation in rural communities.

## Figures and Tables

**Table 1:** Sociodemographic and health characteristics of Latino immigrant respondents (N = 31) in four California counties.

Characteristic	N (%)
**Age in years, mean (SD)**	45 (10)
**Female**	20 (65)
**Marital status**	
Married or living with a partner	24 (77)
Single	3 (10)
Divorced	2 (6)
Refused or unknown	2 (6)
**Household size, mean (SD)**	5 (2)
**Country of birth**	
Mexico	27 (87)
Other or unknown	4 (13)
**Years in the United States, mean (SD)**	24 (11)
**Education level**	
High school or less	22 (71)
Some college or higher	7 (23)
Unknown	2 (6)
**Diagnosed chronic illness** ^1^	7 (23)
**Food security status**	
High	7 (23)
Marginal	8 (26)
Low	8 (26)
Very low	5 (16)
Unknown	3 (9)
**Participation in a government assistance program (ever)** ^2^	
Medi-Cal (California’s Medicaid program)	22 (71)
CalFresh (California’s Supplemental Nutrition Assistance Program/SNAP, formerly food stamps)	15 (48)
Special Supplemental Nutrition Program for Women, Infants, and Children (WIC)	5 (16)

^1^ Chronic conditions listed: asthma, cancer, cardiovascular disease, diabetes, lung disease, obesity. ^2^ Percentages do not add up to 100% as respondents could select all options that applied.

**Table 2:** Experiences of living with food insecurity: findings on compounding stressors and coping strategies with illustrative quotes.

Compounding Stressor	Coping Strategy	Illustrative Quotes
Fear of COVID-19 infection	Reduce frequency of trips to food retailers (e.g., grocery stores, restaurants)	“… because we know there are lots of people at these stores. Well, you have to go out to buy food. That was one step we took to reduce our risk of exposure in places where there are lots of people.” (Pepe, high food security, Fresno County) “We went about two months without buying a hamburger. Sometimes we would just get in the car and go to the drive thru. We did spend a lot less because we were not going out a lot.” (Gladis, high food security, Fresno County) “My wife and I do not go for two reasons—to not spend money and to not expose ourselves to infection.” (Rogelio, low food security, Tulare County)
	Modify food retail trips to reduce risk of infection (i.e., timing, use of protective gear)	“I try to go to the store very early when I know the store will be open very early. I put on my face mask. In the beginning, I also used gloves.” (Lupe, marginally food secure, Merced County)
Economic insecurity and strained household food budgets	Substitute with lower-cost items or brands	“We tried not to buy sodas because we really like soda and juice, and things like that are just not, well not [good]. It is essential to have water, so we bought water instead.” (Rosa, high food security, Merced County) “Now, it’s more potatoes, beans.” (Susana, very low food security, Tulare County)
	Reduce portions	“We are going to purchase less soda—only one bottle and no one can say they want something else like another flavor. Only one for both and one bag of Cheetos for both. We are not buying more and spending more because we can’t right now.” (Roberta, low food security, Tulare County)
	Reduce meat consumption due to high costs	“We were having to budget our food where, one week, we would eat rice and beans. Then the next week, we would have meat.” (Claudia, low food security, Imperial County) “I am not that particular with food. I can use a bag of beans and it lasts like two months. Now that we are more limited, we can’t eat meat every day.” (Victoria, low food security, Fresno County)
	Increase meal preparation	“We rarely eat out—street food, fast food. Rarely. We are always cooking at home because there isn’t enough money.” (Maria, marginally food secure, Merced County) “There’s more days that I am making food at home. Before there were days, like Saturday and Sunday, when we would spend money at a restaurant. Now I prepare food most days of the week.” (Tere, high food security, Imperial County)
	Reduce out-of-home food consumption and restaurant visits	“We used to go sometimes on Saturday or Sunday to eat at a restaurant or get pizza. Each time it was $100 plus because we are so many. We had to stop that.” (Juan, marginally food secure, Fresno County)
Distance from food retailers	Buy in bulk and freeze food	“I freeze more things. I live about an hour away from Costco and am an hour away from Merced [city].” (Castro, marginally food secure, Merced County)

## Data Availability

Select excerpts presented in this study are available upon request in English or Spanish from the corresponding author. The data are not publicly available due to confidentiality and privacy concerns of the participants.
